# Supplementary material for: Outcomes of NAFLD and MAFLD: Results from a community-based, prospective cohort study
Source: PLoS One. 2021 Feb 3;16(2):e0245762. doi: 10.1371/journal.pone.0245762 (PMC7857550; doi:10.1371/journal.pone.0245762)
Supplement: S1 Table — (DOCX) [file pone.0245762.s001.docx]

**Supplementary material**

**Supplementary table 1: New-onset metabolic features and cardiovascular events (with n, % and age and sex adjusted risk ratios) after 7-years (2014), among controls, lean-NAFLD and lean-MAFLD***

| **Outcomes in 2014** | **Controls**  N = 255 | **Lean-NAFLD**  N = 85 | **Lean-MAFLD**  N = 68 | **RR for NAFLD vs controls** | **RR for MAFLD vs controls** |
| --- | --- | --- | --- | --- | --- |
| Incident general obesity | 9/254 (3.5%) | 4/85 (4.7%) | 3/68 (4.4%) | NA | NA |
| Incident central obesity | 39/246 (15.3%) | 10/51 (19.6%) | 8/33 (24.2%) | 1.6  (0.9 – 3.0) | 2.2  (1.1 – 4.4) |
| Incident DM | 31/243 (12.6%) | 14/55 (25.5%) | 10/38 (26.3%) | 1.8  (1.1 – 3.2) | 1.8  (1.0 – 3.5) |
| Incident HTN | 36/218 (16.5%) | 16/52 (30.8%) | 11/35 (31.4%) | 1.7  (1.1 – 2.8) | 1.7  (1.0 – 3.0) |
| Incident TG | 68/231 (29.4%) | 15/43 (34.9%) | 15/30 (50.0%) | 1.1  (0.7 – 1.8) | 1.6  (1.1 – 2.5) |
| Incident low HDL | 68/208 (32.7%) | 28/56 (50.0%) | 25/44 (56.8%) | 1.4  (1.0 – 1.8) | 1.5  (1.1 – 2.1) |
|  |  |  |  |  |  |
| CVD non-fatal and fatal events | 4/253 (1.6%) | 6/81 (7.1%) | 6/65 (9.2%) | 4.4  (1.2 – 1.6) | 5.6  (1.5 – 2.1) |

* Individuals with existing condition at baseline were excluded in the calculation of incidence rates. NA – Risk ratios could not compute due to singular matrix
