# Supplementary material for: Outcomes of NAFLD and MAFLD: Results from a community-based, prospective cohort study
Source: PLoS One. 2021 Feb 3;16(2):e0245762. doi: 10.1371/journal.pone.0245762 (PMC7857550; doi:10.1371/journal.pone.0245762)
Supplement: S1 File — (PDF) [file pone.0245762.s002.pdf]

SriLanka1-

Ragama Health Study
Faculty of Medicine
University of Kelaniya

Individual Number

House Number

Date of the survey

D	D	M	M	Y	Y	Y	Y

Name:

PHM Division

Grama Niladhari Division

Address

Contact Telephone Number:

Number of Household Members

Time of last meal or drink
(excluding water)

Results of the Ultra Sound Scan

Time of taking the blood sample

Height (cm)			.	
Weight (Kg)			.	
Waist (cm)			.	
Hip (cm)			.	

Blood Pressure
(mm Hg)

SBP 1		SBP 2	
DBP 1		DBP 2	

SriLanka1-

Data Collector:

1. Personal Information

	D	D	M	M	Y	Y	Y	Y
**1.2 Date of Birth:**								

1.3 Sex 1 Male
2 Female

1.4 Educational Level

1 No formal schooling
2 Grade 1-5
3 Grade 6-11
4 Qualified G.C.E. O/L
5 Qualified G.C.E. A/L
6 Graduate/ Diplomate
7 Postgraduate
8 Other
(……………………………………………….)

1.5 Occupation:

1 Senior Official and manager
2 Professional
3 Technical and associate professional
4 Clerk
5 Sales and service worker
6 Skilled agricultural and fishery worker
7 Craft and related trades worker
8 Plant and machine operator and assembler
9 Elementary occupations
10 Armed forces and police
11 Unemployed
12 Other
(……………………………………………..)

1.6 Monthly family income

Less than Rs.1500	
Rs.1500-2499	
Rs.2500-4999	
Rs.5000-9999	
Rs.10000-24999	
Rs.25000-49999	
Rs.50000 and above	

1.7 Ethnicity

1 Sinhalese
2 Sri Lankan Tamil
3 Indian Tamil
4 Muslim
5 Malay
6 Burger
7 Other
(…………………………..)

1.8 Religion

1 Buddhism
2 Christianity
3 Hinduism
4 Islam
5 Other (………………………………….)

1.9 Marital status

1 Unmarried
4 Divorced

2 Married **5 Widowed**

3 Separated

2 Household Information

2.1 Kitchen

1 Separate
2 Not separate

2.2 Electricity

1 Yes
2 No

2.3 Drinking water

1 Pipe borne
2 Well
3 Other

1 Boiled
2 Not boiled

Water for other purposes

1 Pipe borne
2 Well
3 Other

2.4 Latrine

1 Yes
2 No

If yes;

1 Own
2 Common

Drainage

1 Within the premises (pit/ septic tank)
2 Out of the premises (sewer system)

2.5 House

1 Own
2 Rented

Number of rooms

Type of House

Roof

1 Tile/ Asbestos
2 Galvanised sheets
3 Cadjans
4 Other

Walls

1 Complete
2 Incomplete

Walls made of:

1 Made with bricks/ cement blocks and plastered
2 Made with bricks/ cement blocks and not plastered
3 Timber
4 Cadjans

5 Other SriLanka1-

2.6 Household assets

			Number/ Square area
**Television**	**1 Yes**	**2 No**	
**Radio**	**1 Yes**	**2 No**	
**Van**	**1 Yes**	**2 No**	
**Car**	**1 Yes**	**2 No**	
**Three-wheeler**	**1 Yes**	**2 No**	
**Motor Bicycle**	**1 Yes**	**2 No**	
**Computer**	**1 Yes**	**2 No**	
**Telephone/ Mobile phone**	**1 Yes**	**2 No**	
**Land**	**1 Yes**	**2 No**	

2.7 Fuel used for cooking

**1 Wood**	**1 Yes**	**2 No**
**2 Kerosene oil**	**1 Yes**	**2 No**
**3 LP Gas**	**1 Yes**	**2 No**
**4 Other**	**1 Yes**	**2 No**

Fuel type used most

SriLanka1-

3 Past medical history

Have you ever been diagnosed of having any of the following conditions by a doctor?

1 Yes
2 No

Disease condition		Current medication		Frequency	Year detected	Confirmed by medical records
Diabetes mellitus		1 Insulin injection<br>2 oral anti-diabetic drugs<br>3 1 and 2 above<br>4 Diet control only<br>5 Other<br>6 Not on treatment				
High Blood Pressure		1 Anti-hypertensive drugs<br>2 Salt reduction<br>3 1 and 2 above<br>4 Others<br>5 Not on treatment				
Hyper-lipidaemia (including hyper-cholesterolaemia)		1 Lipid lowering drugs<br>2 Diet control<br>3 1 and 2 above<br>4 Others<br>5 Not on treatment				

Disease condition		Year diagnosed
Myocardial infarction	1 Yes<br>2 No	
Other Ischaemic Heart Disease	1 Yes<br>2 No	
Stroke	1 Yes<br>2 No	
Cancer	1 Yes<br>2 No	
If yes; What is the primary site of the cancer?		
Lung diseases	1 Yes<br>2 No	
If yes; What is the lung disease?		
Mental illness	1 Yes<br>2 No	
If yes; What is the condition?		

Kidney diseases **1 Yes**
2 No

If yes; What is the kidney disease?

SriLanka1-

4 Family History

4.1 Indicate the following information for your parents. Use following list to indicate the cause of death.

1 Diabetes mellitus **5 Stroke** **9 Other**
2 Ischaemic heart disease **6 Cancer** **10 Unknown**
3 High Blood pressure **7 Mental illness**
4 Hyper-lipidaemia **8 Kidney disease**

**Family member**	**1 Dead**<br>**2 Alive**	**Age at death/**<br>**Current age if alive**	**If dead Cause of Death**
**Father**			
**Mother**			

4.2 How many siblings do you have?

4.3 How many of them are males?

4.4 How many of them are females?

4.5 What is your birth order in the family?

4.6 Have any of your family members been diagnosed as having diabetes mellitus?
1 Yes **2 No**
If no; Go to question 4.7
If yes; Who are the members affected? **1 Yes** **2 No**
Father

Mother

Siblings **How many siblings are affected?**

4.7 Have any of your family members been diagnosed as having ischaemic heart diseases? **1 Yes** **2 No**
If no; Go to question 4.8
If yes; Who are the members affected? **1 Yes** **2 No**
Father

Mother

Siblings **How many siblings are affected?**

4.8 Have any of your family members been diagnosed as having high blood pressure? **1 Yes** **2 No**
If no; Go to question 4.9
If yes; Who are the members affected? **1 Yes** **2 No**
Father

Mother

Siblings **How many siblings are affected?**

Mother

Siblings How many siblings are affected?

4.9 Have any of your family members been diagnosed as having hyper-lipidaemia?
1 Yes 2 No
If no; Go to question 4.10
If yes; Who are the members affected? 1 Yes 2 No
Father

Mother

Siblings How many siblings are affected?

4.10 Have any of your family members been diagnosed as having a stroke?
1 Yes 2 No
If no; Go to question 4.11
If yes; Who are the members affected? 1 Yes 2 No
Father

Mother

Siblings If yes; How many siblings are affected?

4.11 Have any of your family members been diagnosed as having cancer?
1 Yes 2 No
If no; Go to question 4. 12
If yes; Who are the members affected? 1 Yes 2 No
Father If yes; Primary site

Mother If yes; Primary site

Siblings If yes; How many siblings are affected?

What are primary site of the cancer in your siblings?

4.12 Have any of your family members been diagnosed as having mental illness?
1 Yes 2 No
If no; Go to question 5
If yes; Who are the members affected? 1 Yes 2 No
Father

Mother

Siblings How many siblings are affected?

4.13 Have any of your family members been diagnosed as having kidney disease?
1 Yes 2 No
If no; Go to question 5
If yes; Who are the members affected? 1 Yes 2 No
Father
Mother
Siblings **How many siblings are affected?**

SriLanka1-

5 Lifestyle

Alcohol consumption
5.1 Have you ever consumed alcoholic beverages at least once a week for at least one year successive period in your lifetime?
1 No ; Go to question 5.5
2 Yes, but have stopped
3 Yes, but currently taking less than once a week
4 Yes, currently taking at least once a week

5.2 How many years in total have you taken alcohol at least once per week?

5.3 How much alcohol do you take on a day of alcohol drinking?

	Bottles	Units
1 Beer (350 ml/ / ½ pint= 1 unit)		
2 Arrack (50 ml= 1 unit)		
3 Wine (1 glass= 1 unit)		
4 Kasippu (locally brewed illicit alcohol)		
5 Others		

5.4 How many days do you drink per week?

1. **1-2 days a week**
2. **3-4 days a week**
3. **5-6 days a week**
4. **Daily**

Smoking
5.5 Have you ever smoked 100 cigarettes/beedis/cigars or more in your lifetime?
1. **No ; Go to question 5.8**
2. **Yes; but have stopped now**
3. **No; currently smoking less than once a day**
4. **Yes; currently smoking at least once a day**

5.6 How many years in total have you smoked in your lifetime?

5.7 What do you smoke and how much in a day?

	Quantity
1. Cigarettes	
2. Beedi	
3. Cigars	
4. Others (………………………………………………….)	

Dietary habits

5.8 How many cups of tea do you usually drink daily?

1. **None**
2. **Less than daily**
3. **1-2 cups**
4. **3-4 cups**
5. **5 cups or more**

5.9 Do you take any vitamin supplements?

If yes, specify……………………………………………………..

How frequently do you take them?

1. **1-2 days a week**
2. **3-4 days a week**
3. **5-6 days a week**
4. **Daily**

5.10

The following questions are on food items that were regularly consumed by you during the last year. Please indicate the frequency of consumption of each food item in the cage in front of each item according to the classification in the chart below.

In answering:

- **Concentrate only on the food items mentioned in the list.**
- **Include the ingredients used in preparation of food as well.**
- **Exclude the food items that were taken in very small quantities.**

**Less than once a week**	**1**
**Once a week**	**2**
**2-4 times a week**	**3**
**More than 4 times a week**	**4**
**Once a day**	**5**
**More than once a day**	**6**

1	Green leaves (Spinach, Kathurumurunga) and leafy vegetables (cabbage, leeks, leaves of radish, beetroot etc.)	
2	Starchy vegetables (jak, bread-fruit, manioc, sweet-potatoes, potatoes, pumpkin etc.)	
3	All other vegetables (brinjals, okra, beans, snake-gourd, beetroot, knokhol tec.)	
4	Yellow coloured fruits (Papaw, mangoes, lavulu)	
5	Citrus fruits (orange, guava, lemon, nelli, etc.)	
6	All other fruits (bananas, apple, pears, grapes, wood-apple, pineapple, melon etc.)	
7	Full cream milk powder, cow’s milk, pasteurized milk, sweetened milk	
8	Ice-cream, yoghurt, and curd	
9	Other dairy products (cheese, butter, ghee)	
10	Margarines that harden when refrigerated (e.g. Astra margarine)	
11	Margarines that stay soft when refrigerated (Meadow Lee, Flora, Sunflower margarines)	
12	Low fat dairy products (low fat milk powder, low fat cow’s milk, low fat yoghurt)	
13	Egg yolk and Caramel Puddings, Watalappam made with egg yolk	
14	Food made from cereals with bran (brown bread, red rice, food made with red rice flour, food made with kurakkan, whole grain wheat flour, ata flour)	
15	Dhal	
16	Green gram, cow-pea, chick-pea, soya based food as a main meal	
17	Beef, mutton, pork, prawns, crabs, cuttle-fish, squid, and chicken with skin	
18	Processed meat (Sausages, porkies, ham, salami, bacon, meat-balls, hot dogs, burgers)	
19	Deep fried food (fish/ meat rolls, cutlets, patties), bites, wade, cashew, onions	
20	Commercially baked food (pastries, sweets, cakes)	
21	Rotti (vegetable/ fish rotti, koththu roti, coconut rotti, godamba rotti etc.)	
22	Sweetened beverages (tea/ coffee with more than 2 teaspoonfuls of added sugar, sweetened drinks, malt drinks)	

5.11 Physical activity

1. **Recall the heavy work you carried out for at least 10 minutes during the last week (Heavy work is the work that increases your rate of breathing rapidly e.g. lifting heavy weights, digging, cycling at a high speed).**

1 How many days did you do heavy work during the last week? [ ]

If none; go to question 2

2 How much time did you spend doing heavy work in a day?

[ ] **Hours per day**

[ ] **Minutes per day**

2. **Recall the moderate work you carried out for at least 10 minutes during the last week (Moderate work is the work that increases your rate of breathing more than the normal rate e.g. lifting light weights, cycling at a moderate speed).**

1 How many days did you do moderate work during the last week? [ ]

If none; go to question 3

2 How much time did you spend doing moderate work in a day?

[ ] **Hours per day**

[ ] **Minutes per day**

3. **Recall the time that you spent walking for at least 10 minutes continuously during the last week. (at home, at work, from place to place).**

1 How many days did you walk for at least 10 minutes continuously during the last week? [ ]

If none; go to question 4

2 For how long did you walk in a day?

[ ] **Hours per day**

[ ] **Minutes per day**

Not sure [ ]

4. **Recall the time that you spent seated during the last week. (at home, at work, studying, resting, watching TV).**

How much time did you spend seated during the last week? [ ]

[ ] **Hours per day**

[ ] **Minutes per day**

Not sure [ ]

6 Information on general health

6.1 How do you rate your general health?

1. Excellent
2. Good
3. Satisfactory
4. Unsatisfactory
5. Poor

6.2 Have you ever had any blood transfusions? 1 Yes
2 No

6.3 Bowel habits
1 Regular
2 Irregular

6.4 Have you ever been exposed to passive smoking inside home, workplace or in any other place? 1 Yes
2 No

6.5 Have you ever been exposed to indoor air pollutants like wood smoke or smoke from mosquito repellants? 1 Yes
2 No

6.6 Did you notice any change in your body weight during the last 12 months? 1 Yes
2 No

6.7 How do you rate your mental status?

1. Sad / depressed
2. There is a lack of interest for generally satisfying activities
3. Normal/ No difference

6.8 Life events

Event	1 Yes<br>2 No	How long ago (months)
Death of spouse		
Divorce		
Separation form spouse		
Imprisonment		
Death of an immediate relative		
Illness or an accident		
Serious illness (e.g. cancer/ cardiac surgery/ heart attack, etc.)		
Loss of job		

6.9 How is your sleep pattern?

1. Excellent
2. Good
3. Satisfactory
4. Unsatisfactory

5. Poor

SriLanka1-

ONLY FOR FEMALES

7 Reproductive history

7.1 What was your age at menarchae [ ][ ] years

7.2 Has menopause occurred? 1 Yes / 2 No [ ] If no; Go to 7.3

If yes; What was your age at menopause? [ ][ ] years

7.3 If you currently have menstrual cycles , are they regular? [ ]

1. Regular
2. Irregular

7.4 Length of the menstrual cycle (days) [ ][ ]

7.5 Duration of menstruation (days) [ ][ ]

7.6 Last regular menstrual period D D M M Y Y

D	D	M	M	Y	Y

7.8 Obstetric History

Parity	Result<br>1 Live birth<br>2 Still birth<br>3 Abortion	Mode of delivery<br>1 NVD<br>2 LSCS<br>3 Forceps<br>4 Vaccum	Age at birth of child	Birth Weight (g)	Duration of breast feeding (months)
1					
2					
3					
4					
5					
6					
7					
8					
9					
10					

7.9 Contraception

Method	1 Past user<br>2 Current user	Duration (months)
1 Pills		
2 Injectables		
3 IUCD		
4 Norplant		

7.10 Have you ever had surgeries of following organs?
1 Yes
2 No

1 Ovaries	
2 Breast	
3 Uterus	
