# Supplementary material for: Outcomes of NAFLD and MAFLD: Results from a community-based, prospective cohort study
Source: PLoS One. 2021 Feb 3;16(2):e0245762. doi: 10.1371/journal.pone.0245762 (PMC7857550; doi:10.1371/journal.pone.0245762)
Supplement: S2 File — (PDF) [file pone.0245762.s003.pdf]

Questionnaire 1 – Telephone Interview by MBBS Graduate

Name :………………………………………………………………….. Tel :……………………………

Age :……............................. Date of Birth :………………………………..

Sex : M ☐ F ☐

Address :…………………………………………………………………………………………………..

RHS Number :……………………………………..

1. Is the participant alive? Yes ☐ No ☐

   (If Yes – Go to Question 2)

   If No - date of death :……………………………………………………..

   cause of death (from DC) :……………………………………………………..

   cause of death not known :……………………………………………………..

   If cause of death not clear - Needs Home Visit

2. From 2007 up to now has the participant had any of the following?

   a) non-fatal myocardial infarction (heart attack) - Yes ☐ No ☐

      (If Yes go to Question 3)

   b) non-fatal stroke - Yes ☐ No ☐

      (If Yes go to Question 4)

   c) coronary artery bypass surgery (bypass) – Yes ☐ No ☐

   d) percutaneous transluminal coronary angiography (stent) - Yes ☐ No ☐

3) (If Yes to a) – Clarify whether clinically significant – Yes ☐ No ☐

[Clinically significant chest pain = requiring hospital admission for >24 hours and requiring special treatment (streptokinase, heparin, cardiology referral with or without echocardiography and/or referral for coronary intervention)]

If Yes fill relevant table (2-5) in questionnaire 2

4) (If Yes to b) - Clarify whether clinically significant – Yes ☐ No ☐

[Clinically significant neurological deficit = sudden onset neurological deficit lasting >24 hours, requiring a CT scan of the brain and/or management in a specialized Stroke Unit]

If Yes fill relevant table (2-5) in questionnaire 2

5) Further details needed based on 1-4 - Yes ☐ No ☐

If Yes - Needs Home Visit

Questionnaire 2 – Home Visit by MBBS Graduate

Name :………

Age :………………………   Date of Birth :………………………………………………………

Sex : M ☐ F ☐

Address :………………………………………………………………………………………………………..

RHS Number :…………………………………………………………………………………………….....

1) **Death of participant between 2007-2014**

Date of Death – …………………………………..

Cause of Death – ………………………………..

Previous Non-Fatal Events – Yes ☐ No ☐

(If Yes go to relevant Table below)

2) **Non-fatal MI**

Date			
Hospital			
Diagnosis			

3) **Non-fatal Stroke**

Date			
Hospital			
Diagnosis			

4) **Coronary artery bypass surgery**

Date			
Hospital			

5) **Percutaneous transluminal coronary angiography**

Date			
Hospital			
Diagnosis			
